# Synthesis, Spectroscopic, Chemical Characterizations, Anticancer Capacities against HepG-2, Antibacterial and Antioxidant Activities of Cefotaxime Metal Complexes with Ca(II), Cr(III), Zn(II), Cu(II) and Se(IV)

**DOI:** 10.3390/antibiotics11070967

**Published:** 2022-07-19

**Authors:** Eman H. Al-Thubaiti, Samy M. El-Megharbel, Bander Albogami, Reham Z. Hamza

**Affiliations:** 1 Biotechnology Department, College of Sciences, Taif University, Taif-P.O. Box 11099, Taif 21944, Saudi Arabia; i.althubaiti@tu.edu.sa; 2 Chemistry Department, College of Sciences, Taif University, Taif-P.O. Box 11099, Taif 21944, Saudi Arabia; s.megherbel@tu.edu.sa; 3 Biology Department, College of Sciences, Taif University, Taif-P.O. Box 11099, Taif 21944, Saudi Arabia; b.boqami@tu.edu.sa

**Keywords:** metal complexes, spectroscopic studies, antioxidant capacities, hepatic functions, cancer cells, cefotaxime

## Abstract

In this study, metal cefotaxime complexes of Ca(II), Cr(III), Cu(II), Zn(II), and Se(VI) were synthesized and characterized by elemental analysis, conductance measurements, IR, electronic spectra, magnetic measurements, ^1^HNMR, and XRD, as well as by scanning electron microscopy (SEM) and transmission electron microscopy (TEM). The lower values for molar conductance refer to the nonelectrolyte nature of the complexes. The FTIR and ^1^H-NMR spectra for the metal complexes of cefotaxime proved that the free cefotaxime antibiotic ligand acted as a monoanionic tridentate ligand through the oxygen atoms of lactam carbonyl, the carboxylate group, and the nitrogen atoms of the amino group. From the magnetic measurements and electronic spectral data, octahedral structures were proposed for the Cr(III) and Se(VI) complexes, while the Cu(II) complex had tetragonal geometry. This study aimed to investigate the effects of cefotaxime and cefotaxime metal complexes on oxidative stress using antioxidant assays including DPPH, ORAC, FARAB, and ABTS, a metal chelation assay, as well as the inhibition of the viability of cancer cells (HepG-2). Regarding the antibacterial activity, the cefotaxime metal complexes were highly effective against both *Bacillus subtilis* and *Escherichia coli*. In conclusion, the cefotaxime metal complexes exhibited highly antioxidant activities. The cefotaxime metal complexes with Zn and Se inhibited HepG-2 cellular viability. Thus, the cefotaxime metal complexes elicited promising results as potent antioxidant and anticancer agents against HepG-2, with potent antibacterial activities at a much lower concentration.

## 1. Introduction

**Figure 1:**
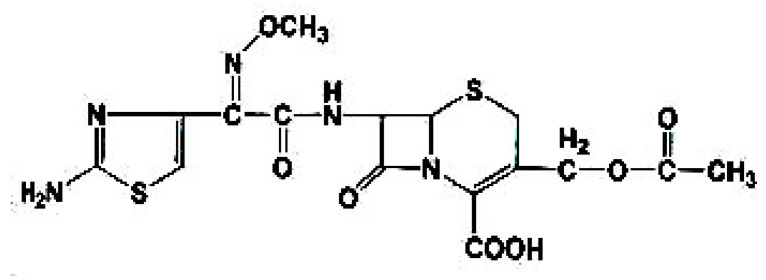
The structure of cefotaxime.

Infectious diseases resulting from bacteria are considered a major health problem worldwide, part of which can be attributed to the rapidly increasing resistance to existing antimicrobial drugs. For treating pathogenic multidrug-resistant bacterial strains, developing new antimicrobial compounds is vital. The antibiotic drug, cefotaxime, shown in Figure 1, is considered a third-generation cephalosporin; the resistance to it is due to the inability of cefotaxime to reach its target sites, to alter the binding of penicillin with proteins, which is the main target of the cephalosporins; in addition, β-lactamases (bacterial enzymes) deactivate cephalosporin [1].

The hydrolysis of third-generation cephalosporins has a higher resistance to β-lactamases formed by some Gram-negative bacterial strains than that of the first generation of cephalosporins. Cefotaxime is used in the treatment of infections of the respiratory tract, meningitis, and septicemia [2].

For the action of synthetic and natural metalloantibiotics, metal ions play an important role, as they are involved in specific interactions with some biomolecules, such as nucleic acids, proteins, and membranes [3].

The mode of chelation of cefotaxime occurs through the nitrogen of the NH_2_ group, the lactam carbonyl group, and carboxylate; thus, it acts as a tridentate ligand [4]. The reactions of cefotaxime with some transition metal ions, such as Cr(III), Mn(II), Fe(III), Co(II), Ni(II), Cu(II), and Zn(II), have been studied [5] and elucidated using different spectroscopic tools, including IR, electronic, magnetic susceptibility, and ESR spectra. The obtained data confirmed that the cefotaxime may act as a mono, di, tri, and tetra-dentate via the lactam carbonyl, carboxylic, or amide carbonyl groups and the N atom of the thiazole ring. New cefotaxime complexes of the general formula [ML2(H_2_O)_2_], where M = Co(II), Ni(II), Cu(II), and Zn(II), while L represents the Schiff base ligand, were prepared by reaction of cefotaxime with salicylaldehyde in an ethanolic medium.

The characterizations of the complexes’ structures were elucidated by conductance measurements, analyses of C, H, and N, magnetic investigations, and IR and UV spectroscopy [6]. The low conductance values referred to nonelectrolyte types of complexes. According to the electronic spectra and magnetic data, the Co(II), Ni(II), and Zn(II) complexes had an octahedral geometry, while the Cu(II) complex had tetragonal geometry. Many antibiotic drugs have modified toxicological and pharmacological properties with the presence of these drugs in form of metal complexes. Copper(II) is considered to be the most common metal in this field, which plays a role in the treatment of diseases such as gastric ulcers and rheumatoid arthritis [7,8,9,10].

These data encourage the study of the biological activity and the coordination chemistry of antibiotic drugs with transition metal ions to investigate the binding modes in the solid state. In continuation of the metal interactions examined in experimental work with derivatives of β-lactam [11,12,13,14], we studied the preparation and spectroscopic characterization of metal cefotaxime complexes. The major goal of the present work was to assess the antimicrobial activity of complexes of cefotaxime prepared with metal ions of calcium, chromium, copper, zinc, and selenium.

## 2. Experimental Methods

### 2.1. Chemical Reagents

All reagents used in this work, salts of metals, chemical solvents, and the cefotaxime antibiotic drug purchased from Sigma Aldrich were of reagent grade.

### 2.2. Synthesis of Cefotaxime Metal Complexes

The complexes of: [M (cefotax)Cl]·nH_2_O (M = Ca(II), n = 1, Zn(II), n = 2 and Cu(II), n = 3), [Cr (cefotax)(H_2_O)Cl_2_]·H_2_O, and [Se (cefotax) Cl_3_]·2H_2_O were synthesized by mixing of (1 mmol) from cefotaxime dissolved in 25 mL MeOH and (1 mmol) from metal chlorides of Ca(II), Cr(III), Zn(II), Cu(II), and Se(IV) dissolved in 20 mL distilled H_2_O, then reaction mixture was stirred at room temperature for ca. 6 h, and left to stand overnight. The produced precipitates were filtered, washed using mixture of (MeOH distilled H_2_O), and finally dried. The analytical data for isolated solid complexes, Table 1, depict the formation of complexes with stoichiometry of 1:1.

### 2.3. Instruments

For free cefotaxime ligand and its metal complexity, IR spectra were recorded as potassium bromide disks using infrared Bruker spectrophotometer ranging from 400–4000 cm^−1^. UV–vis. spectra were recorded using DMSO solvent within range 800–200 nm using a UV2 Unicam UV/Vis Spectrophotometer fitted with a quartz cell of 1.0 cm path length. C, H, N, and S were performed by the microanalysis unit at Cairo University, Egypt, using a Perkin Elmer CHN 2400 instrument. With concentration of 10^−3^ M in DMSO solvent, conductivity measurements for cefotaxime metal complexity were measured using HACH conductivity meter model. Magnetic measurements were determined at room temperature using “Johnson Matthey” susceptibility balance by using Hg (II) tetrathiocyanato-cobaltate(II) as a calibrant. Spectra of ^1^H NMR were investigated using “Bruker AM-500 NMR spectrometer”, using TMS and deuterated DMSO as solvent. Scanning electron microscopy (SEM) images were taken with Joel JSM-6390 equipment, with an accelerating voltage of 20 KV. The X-ray diffraction patterns were recorded on X ‘Pert PRO PAN analytical X-ray powder diffraction, target copper with secondary monochromate. The images of transmission electron microscopy (TEM) were performed using JEOL 100 s microscopy. The concentrations of metals in all experiments were performed using Flame atomic absorption spectroscopy instrument (Perkin Elmer Analyst 400, Waltham, MA, USA).

### 2.4. Antioxidant Activity

#### 2.4.1. ORAC Assay

For cefotaxime and its metal complexes, the antioxidant activity was assessed based on Liang et al. [15]. For each, 10 µL of the cefotaxime and/or cefotaxime metal complexes were incubated for about 10 min with fluorescein at 37 °C. Subsequently, 70 µL of freshly prepared 2,2′-Azobis (2-amidinopropane) dihydrochloride (AAPH) (300 mM) were added immediately to each well. Fluorescein measurement (485 EX, 520 EM, nm) was continued for 1 h (40 cycles, each one 90 s). Data are represented as means (n = 3) ± SD and the antioxidant effect of the compound/extract was calculated as µM Trolox equivalents by substitution in the linear regression equation y = 4275.8x + 262,311.

#### 2.4.2. The Test of Metal Chelation

The test of metal chelation was performed based on Santos et al. [16]. In total, 20 µL of freshly prepared FeSO_4_ (0.3 mM) were mixed with about 50 µL of either cefotaxime and/or metal complexes in well plates before adding 30 µL of ferrozine. Next, all the plates were incubated for about 10 min at 37 °C. Any decrease in the intensity of color was measured at 562 nm. Data are represented as means ± SD according to the following equation:

percentage inhibition = ((average absorbance of blank − average absorbance of the test)/(average absorbance of blank)) × 100.(1)

#### 2.4.3. The Test of DPPH

The antioxidant capacities of cefotaxime and its metal complexes were measured by 100 µL of freshly prepared DPPH reagent (0.1% in methanol) (2,2-diphenyl-1-picryl-hydrazyl-hydrate) free-radical assay carried out according to Boly et al. [17]. In total, 100 µL of the freshly prepared reagent were dissolved in methanol and then were added to cefotaxime and its metal complexes. The chemical reactions were then incubated for about 1/2 h in dark at about 37 °C. The resulting reduction in DPPH color intensity was measured at 540 nm. Data are represented as means ± SD according to the following equation:

percentage inhibition = ((average absorbance of blank − average absorbance of the test)/(average absorbance of blank)) × 100.

#### 2.4.4. ABTS Assay

The ABTS assay of cefotaxime and its metal complexes was carried out according to Arnao et al. [18]. In total, 192 mg of ABTS reagent were immediately dissolved in 1 mL dist. H_2_O and transferred to 50 mL volumetric flask; the volume was then completed with distilled water. One mL of the previous solution was added to 17 µL of K_2_S_2_O_8_ (140 mM) and the mixture was left in the dark for 24 h. Next, 1 mL of the reaction mixture was completed to 50 mL with methanol to obtain the final ABTS dilution used in the assay; the chemical reaction was incubated at 37 °C for 1/2 h in dark. At the end of incubation time, the decrease in ABTS color intensity was measured at 734 nm. Data are represented as means ± SD according to the following:

percentage inhibition = ((average absorbance of blank − average absorbance of the test)/(average absorbance of blank)) × 100.

#### 2.4.5. FARAB Assay

FARAB test was carried out based on Benzie et al. [19], on microplates. We used a freshly prepared TPTZ reagent (300 mM acetate buffer (PH = 3.6), 10 mM TPTZ in 40 mM HCL, and 20 mM FeCl_3_, in a ratio of 10:1:1 *v/v/v*, respectively. In total, 190 μL from the freshly prepared TPTZ reagent were mixed with 10 μL of the sample on 96-well plates (n = 3). The reaction was incubated for 1/2 h in dark at 37 °C. The resulting blue color was measured at 593 nm. Data are presented as mean ± SD.

### 2.5. Cell Culture

HepG2 was freshly obtained from Scientific Inc., Cairo, in Egypt. The cells were kept in DMEM medium with streptomycin, penicillin, and fetal bovine serum in a 5% CO_2_ atmosphere at about 37 °C. The cell lines were authenticated by STR analysis using the Gene Print 10 system (Promega corporation, Madison, WI, USA) [20].

#### 2.5.1. Cytotoxicity Assay

HepG2 cells were maintained in a DMEM medium supplemented with streptomycin, penicillin, and fetal bovine serum in 5% CO_2_ atmosphere at about 37 °C. The cell viability was assessed by SRB assay. Aliquots of about 100 μL of the cell suspension were seeded in well plates and immediately incubated in a complete medium for 24 h. The cells were then treated with another medium containing the drugs at various concentrations. After 3 days of exposure to the cefotaxime and cefotaxime metal complexes, the cells were fixed by replacing the medium with 10% TCA and incubated for 1 h at about 4 °C. The TCA solution was then removed, and the cells were washed 5 times with distilled H_2_O. Aliquots of the SRB solution were added and incubated at 37 °C in the dark for about 10 min. The well plates were then washed 3 times with acetic acid (1%) and air-dried. Next, 150 μL of TRIS were added to dissolve the protein-bound SRB stain. The absorbance was measured at 540 nm using a “BMGLABTECH^®^-FLUOstar” Omega microplate reader (Ortenberg, Germany) [21].

#### 2.5.2. Cytotoxic Activity (IC50 Determination)

The inhibitory concentration (IC50) of cell viability was determined by using MasterPlex 2010 software. The percentage of viable cells was plotted as a function of concentration to obtain the IC50 values. Three independent experiments were performed for all assays. The mean value from the triplicate experiments was calculated, and the results were reported as mean (±standard deviation). Control percentage was considered as 100%.

Inhibition percentage (%) was measured using the following equation:

Inhibition percentage (%) = (OD of control) − (OD of sample)/(OD of control) × 100

where OD = optical density.

IC50 is the tested compound concentration that inhibits or kills 50% of cells and is obtained by plotting the inhibition percentage versus the test-compound concentration.

### 2.6. Antibacterial Activity against Bacillus Subtilis (ATCC 6633) and Escherichia coli (ATCC 8739)

Discs of *Bacillus subtilis* ATCC 6633 and *Escherichia coli* ATCC 8739 were inoculated into tryptic soy-broth medium and immediately incubated at about 30 °C for (18–24 h). Using a fresh-culture agar plate, a scoop from each broth was gently streaked into agar medium and then incubated at 30 °C. A sterile normal physiological saline (0.9%) was freshly prepared by inoculating about 3 to 4 colonies; the suspension was adjusted to achieve a turbidity equivalent to a 0.5 McFarland standard of each organism using a DensiCHEK^©^ optical device (Anglum Road, Hazelwood, St. Louis, MO, USA). After convenient growth, 1.0 mL Muller Hinton Broth was inoculated into all testing wells (except for the first well). Next, 2 mL from cefotaxime, cefotaxime/Se, cefotaxime/Zn, cefotaxime/Cr, and cefotaxime/Cu were directly inoculated into the first well (without dilution); next, 1 mL was aspirated and transferred to the next well, previously filled with 1.0 mL Muller Hinton Broth. Subsequently, 1 mL was aspirated using a new tip and added to the next 1 mL broth. Next, 1 mL of the prepared inoculum was added to each well (1:2 dilution). This resulted in a final concentration of 5.0 × 10^5^ CFU/well. A further 1 mL from the *Bacillus subtilis* and *Escherichia coli* suspensions was cultured. The growth-control well containing broth, without cefotaxime or its complexes, was added to each sample/plate. A negative-control well containing only the broth without the tested cefotaxime and its novel metal complexes and without bacteria was added to each sample plate [22]. All well plates were then incubated at about 30 °C for 24 h. After incubation, the well plates were removed from the incubator and placed on a dark surface to check for growth. All growth-control wells yielded turbid growth (T), and all negative control wells were found to be clear, showing the validity of the test [23].

### 2.7. Statistical Analysis

The results are presented as mean ± SE using Statistical Package for Statistical Sciences (SPSS) software version 27 (IBM Corp: Armonk, NY, USA, 2020) and Open Epi version 2.3.1. One-way ANOVA and post hoc power were used to analyze the data. A value of *p* < 0.05 was considered significant (using three replicates).

## 3. Results

### 3.1. Microanalytical and Physical Data

The analyses of C, H, N, S, and M^+n^ were convenient due to the stoichiometry of metal: the ligand was 1:1 for all cefotaxime metal complexes (Table 1). All complexes are solids stable in air, soluble in DMF and DMSO. The values of the molar conductivity, measured at room temperature in DMSO, were in agreement with the range of non-electrolytic character [24], suggesting that Cl^−^ ion was chelated with the metal ions inside the complex sphere.

### 3.2. Infrared Spectra

**Figure 2:**
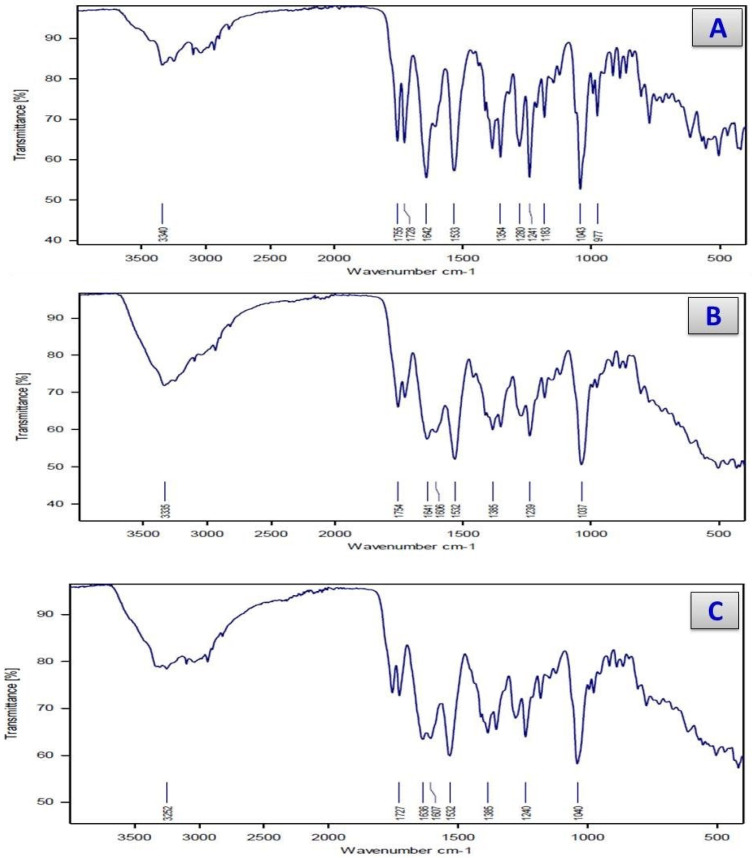

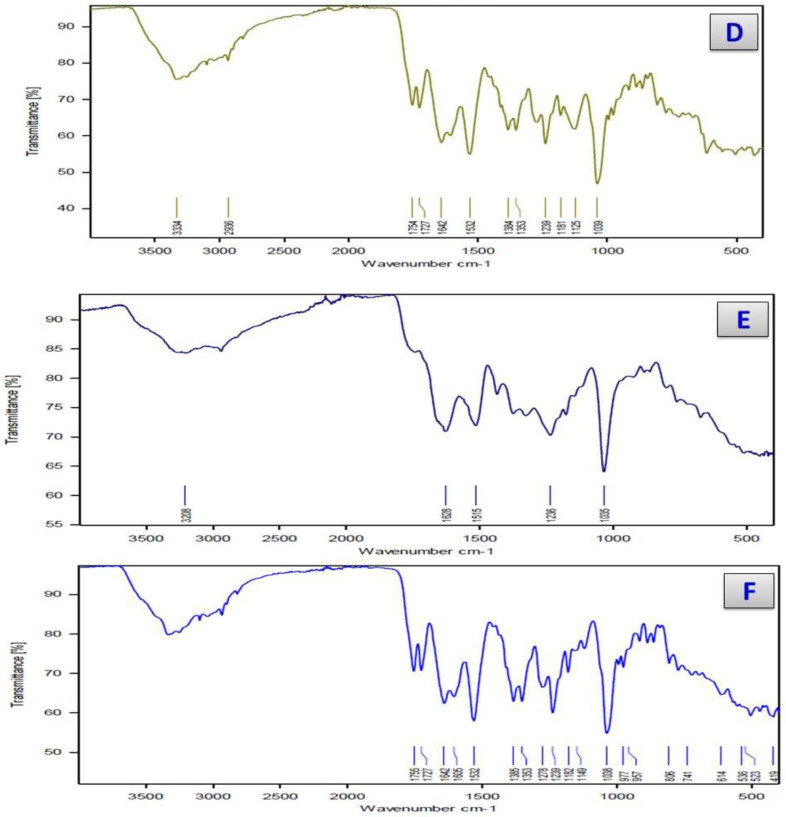
Infrared spectra of (**A**) Cfotax, (**B**) cefotax/Ca, (**C**) cefotax/Cu, (**D**) cefotax/Cr, (**E**) cefotax/Zn, and (**F**) cefotax/Se.

The infrared spectral data for the cefotaxime and its metal complexes were similar. The main IR bands are in Table 2 and Figure 2. The obtained spectral data had the same absorption pattern, in the range of 3500–2800 cm^−1^, which can be attributed to the stretching vibrations of (O-H), (N-H), aromatic (C-H), and aliphatic (C-H) (Guzler and Germlich, 2002) that were observed in the spectra of the free cefotaxime and its metal complexes, with some shifts in the band frequencies owing to changes in the electronic density distribution for the aromatic rings and the main attached functional groups that occurred after the chelation of the metals. A band appeared in the IR spectrum of the cefotaxime at 1775 cm^−1^, which was characterized as (C=O) lactam, while the (C=O) amide and ester bands overlapped at 1642 cm^−1^; for the cefotaxime metal complexes, bands ranging from 1715–1740 and 1630–1650 cm^−1^ appeared.

Based on these data, it can be concluded that the cefotaxime free ligand coordinated through the oxygen atom of the (C=O) lactam group rather than that of the (C=O) amide and ester group, since the bands of the (C=O) lactam group were shifted toward fewer frequencies (35–60 cm^−1^) related to the value of the cefotaxime free ligand, while there was no significant shift for the overlapping bands of the ester and amide carbonyl.

The mode of chelation of the carboxylate group of cefotaxime ligand can bind as a monodentate or a bidentate, which leads to shifts in the stretching vibrations of antisymmetric and symmetric motions [25]. The main difference between νas (COO^−^) and νs (COO^−^): ∆ν = νas (COO^−^)–νs (COO^−^) produced values > 200 cm^−1^, suggesting monodentate chelation for the (COO^−^) group. The IR spectra for the cefotaxime complexes presented bands in the ranges of 640–614 cm^−1^ and 536–523 cm^−1^, which may have been due to the ν(M-O). These bands are not observed in the cefotaxime spectrum. The presence of new bands in the range of 470–495 cm^−1^ in the spectra of the cefotaxime complexes may be attributed to the ν(M-N) (from the NH_2_ group). These bands are absent from the cefotaxime free ligand, suggesting cefotaxime’s coordination character as a chelating agent with a tridentate monoanionic character [26]. The chelation of the NH_2_ group with metal ions was not the only explanation for these bands. When the nitrogen atom of the C=N-OCH_3_ group could not chelate with the metal ions in solid complexes due to steric strains, the coordination of this nitrogen atom along with the carboxylate and lactamic carbonyl groups could not occur. This can be attributed to the contribution of the -NH_2_ group to the coordination process. Finally, based on the above data from the IR spectra for the cefotaxime complexes, the proposed mode of chelation of cefotaxime with metal ions in nature through the lactam carbonyl, carboxylate group and amino group is tridentate in nature.

### 3.3. UV–Vis Spectra

The UV–vis spectra and its assignments for the free-ligand cefotaxime and its metal complexes in the DMSO solvent are presented in Table 3. By comparing the spectra for the cefotaxime base ligand and its metal complexes we deduced that cefotaxime has three absorption maxima, at 273, 320, and 350 nm. The band that appeared at 273 nm was assigned to π → π* transitions due to the organic moiety, while the band that appeared at 320 nm was assigned to intraligand of the π → π* transitions within the heterocyclic moieties [27,28]. The band at 350 nm was assigned to transition of the intraligand of sulphur atoms of the n → π* type, which agreed with the literature data [27,29]. Based on the obtained data for the cefotaxime complexes, the bands due to the S atoms did not shift, suggesting that the S atom did not participate in the chelation process. The calcium(II), chromium(III), zinc(II), and selenium(IV) complexes showed weak bands at (285,322 and 391 nm), (280,327 and 405 nm), (288,331 and 407 nm), and (286,330 and 410 nm), which can be assigned to the π → π* and n → π* transitions.

### 3.4. H-NMR Spectra

**Figure 3:**
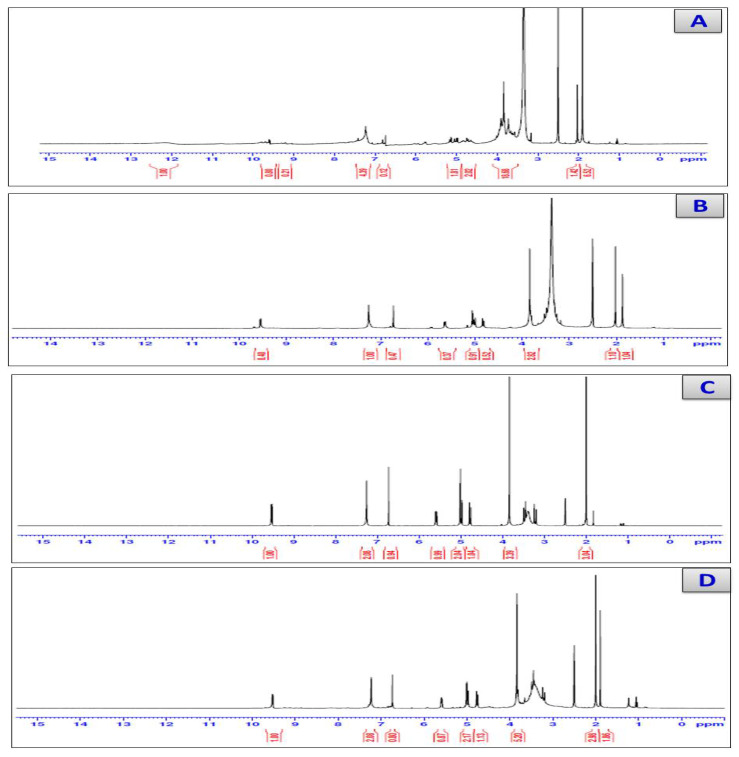
^1^H-NMR spectra of (**A**) Cfotax, (**B**) cefotax/Ca, (**C**) cefotax/Zn, and (**D**) cefotax/Se.

For the cefotaxime free ligand, the spectrum of the ^1^H NMR (Table 4 and Figure 3) had two single peaks due to the CH_3_ groups that appeared at 1.12 and 1.91 ppm. There were three double peaks for the CO-CH and N-CH on the β-lactam ring and the NH at 4.956, 5.770, and 9.534 ppm, respectively. The S-CH_2_ and CH_2_-O groups presented peaks at 3.127 and 4.174 ppm, respectively. The ^1^H NMR spectra for the calcium (II), zinc(II), and Se(IV) cefotaxime complexes changed slightly compared with the cefotaxime, where, as expected, signals appeared downfield due to the occurrence of chelation between the cefotaxime ligand and the metal ions, which resulted in increasing conjugation. On the other hand, the signals of the NH_2_ group observed at 7.459 ppm shifted to 7.249, 7.255, and 7.229 ppm for the Ca(II), Zn(II), and Se(IV), which means that the NH_2_ participated in the coordination process. For the Se (IV) cefotaxime complexity, the band that appeared at 3.831, 3.836, and 3.808 ppm for the calcium, zinc, and selenium complexes was assigned to water molecules, which were not present in the ^1^H NMR spectrum of the cefotaxime free ligand. Upon comparison with the free-ligand cefotaxime, the signal that appeared at 12 ppm was attributed to the protons in the COOH group. This signal peak disappeared in the spectra of the calcium, zinc, and selenium cefotaxime complexes, confirming the chelation of the cefotaxime ligand with the calcium(II), zinc(II), and Se(IV) ions through the deprotonated COOH group. Based on these data, we can conclude that cefotaxime behaves as a monoanionic tridentate ligand through the carboxylic, amino, and carbonyl beta lactam ring group.

### 3.5. The Magnetic Measurements

According to the magnetic susceptibility values, the corrected magnetic moments were determined by using Pascal’s constants. For the cu (II) complex at room temperature, the value of the magnetic moment was 2.03 B.M. which is adequate with systems of d^9^ with one unpaired electron. The observation that this was the highest magnetic-moment value found for the cu(II) complex was based on the fact that spin-orbital coupling in the ion can mix with the ground state, confirming that there is no orbital momentum with higher levels of identical multiplicity, leading to small orbital contribution, which was in accordance with square planar or distorted tetrahedral geometries [30,31,32]. However, the presence of impurities cannot be discarded. The magnetic moment for the chromium(III) cefotaxime was 3.58 B.M., as calculated for system d^3^ with three unpaired electrons, in accordance with octahedral geometry [33]. The experimental magnetic susceptibility value for the Se(IV) was 5.40 B.M., which was in agreement with the calculated value for octahedral geometry [33].

### 3.6. Thermogravimetric Analyses

**Figure 4:**
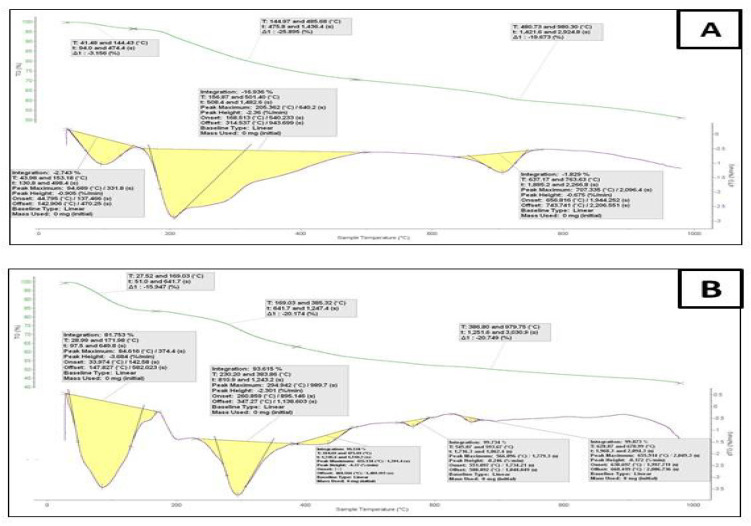
Thermogravimetric analysis of (**A**) cefotax/Ca and (**B**) cefotax/Cu.

The thermogravimetric and differential thermogravimetric analyses (TGA-DTG) for the Ca^+2^ and Cu^2+^ cefotaxime complexes were carried out up to 1000 °C, as shown in Figure 4. It was observed that the Cefotax/Ca and Cefotax/Cu metal complexes induced losses in weight that were up to 169 °C and 144 °C, owing to the loss of water molecules outside the chelation sphere, which suggested that H_2_O molecules were present outside the sphere of chelation. The thermal analysis curves for the Ca(II) and Cu(II) cefotaxime complexes showed losses in weight of up to 980 °C, suggesting that the losses in weight for the Ca(II) and Cu(II) cefotaxime complexes corresponded to the evaporation of the cefotaxime ligand and chlorine atom. The thermal decomposition steps for the divalent calcium and copper complexes were five and three, respectively. As shown on the thermogram of the calcium and copper cefotaxime complexes, CaO and CuO were the most stable final products.

### 3.7. X-ray Diffraction and Scanning and Transmission Electron Microscopy

**Figure 5:**
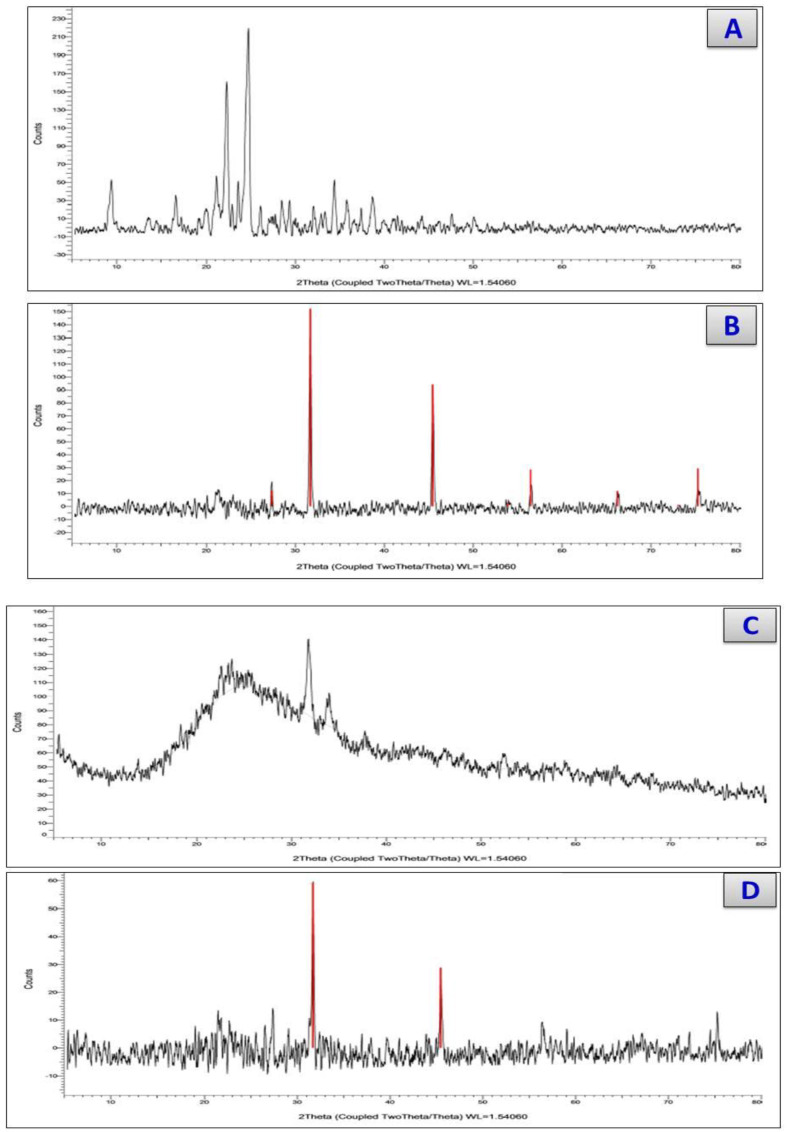

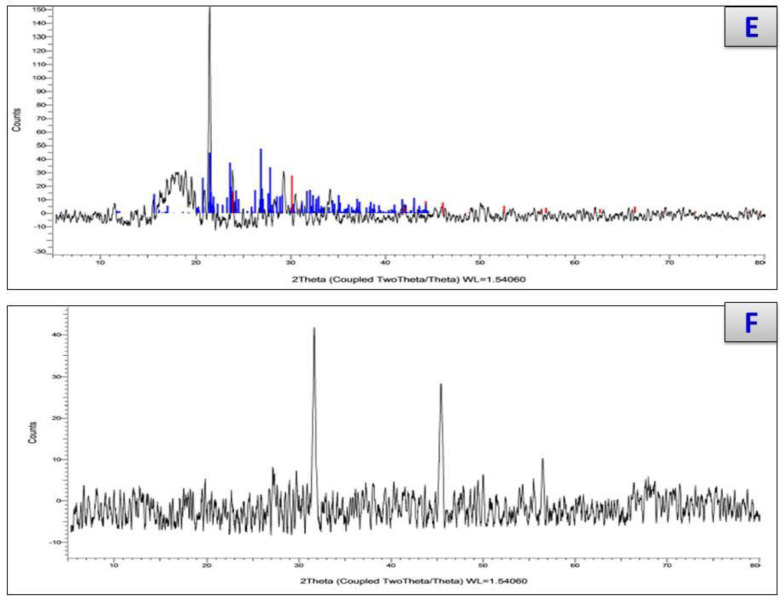
XRDs of (**A**) Cfotax, (**B**) cefotax/Ca, (**C**) cefotax/Cu, (**D**) cefotax/Zn, (**E**) cefotax/Se, and (**F**) cefotax/Cr.

The X-ray diffraction was carried out to confirm the structures of the cefotaxime complexes. For the cefotaxime complexes, the diffractograms produced are shown in Figure 5. The XRD diffractograms confirmed that all the cefotaxime complexes had formulating structures. Many different peaks were well defined at 2θ = 10, 17, 24, 26, 34, 35, 37, and 39 for the Cefotax, 28, 32, 45, 57, and 67 for the Cefotax/Ca (JCPDS = 9–432), 32 and 35 for the Cefotax/Cr (JCPDS = 33–0664), 32, 46, and 71 for the Cefotax/Zn (JCPDS = 36–1451), 21, 24, 29, and 30 for the Cefotax/Cu, and 32, 46, and 57 for the Cefotax/Se. It can be observed that the pattern for free ligand cefotaxime is different from that of its metal complexes, and this can be used as an indication of a well-defined formation and complete crystalline structure. The solid cefotaxime complexes were characterized by X-ray diffraction at room temperature using Cu Kα radiation. The diffraction characterizations of the prepared complexes were detected between 10° and 80°. The crystalline sizes of the synthesized complexes were determined by using the Scherrer equation [34] D = kλ/βCosθ, where k is a constant (0.94), λ is the wavelength (0.154 nm), and β is a full-in-width-at-half-maxima peak. The extent of the crystallinity of the cefotaxime complexes was calculated for the divalent cefotax/Ca, cefotax/, Cu, cefotax/Zn, trivalent chromium, and tetravalent selenium with values of 57 nm. 14 nm, 16 nm, 10 nm, and 15 nm respectively.

**Figure 6:**
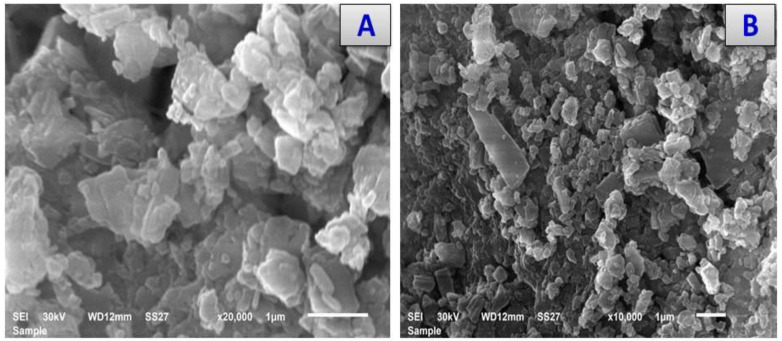

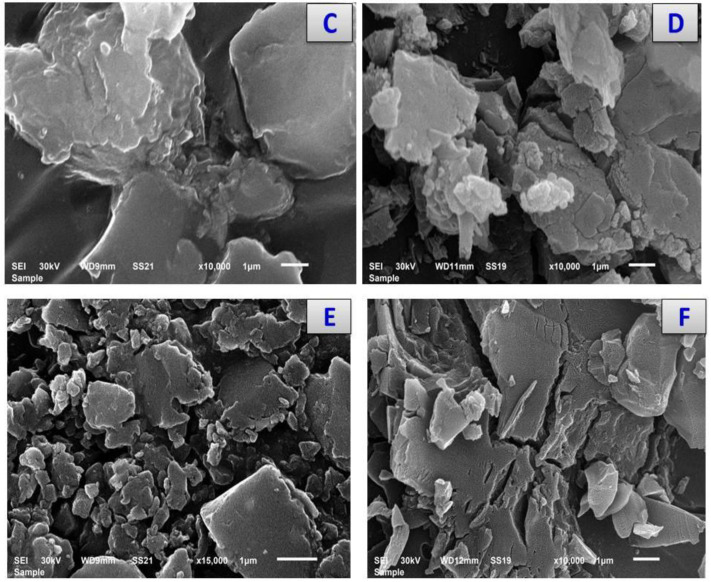
Scanning electron microscopy of (**A**) Cfotax, (**B**) cefotax/Ca, (**C**) cefotax/Cr, (**D**) cefotax/Cu, (**E**) cefotax/Zn, and (**F**) cefotax/Se. (1 µm).

The SEM images offer an explanation for the morphological surfaces of the Ca (II), Cr (II) Cu (II), Zn (II), and Se (VI) cefotaxime complexes that are shown in Figure 6, which give an impression of the SEM images of the NPs formed by the chelation process. The images scanned for the cefotax/Ca, cefotax/Cr, cefotax/Cu, cefotax/Zn, and cefotax/Se by using SEM allowed a full examination of the morphological phases. The SEM images, which were homogenous in size, show that the NPs of the prepared cefotaxime complexes were very close to each other. This can be attributed to the aggregation shape and size.

**Figure 7:**
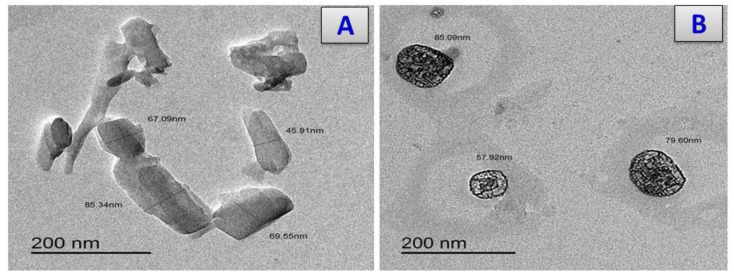

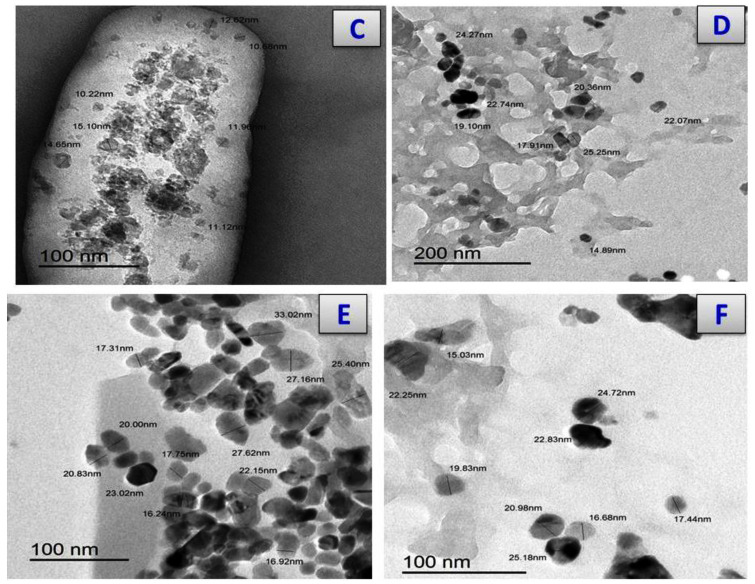
TEM images of (**A**) Cfotax, (**B**) cefotax/Ca, (**C**) cefotax/Cr, (**D**) cefotax/Cu, (**E**) cefotax/Zn, and (**F**) cefotax/Se.

The TEM images for the prepared cefotaxime complexes are presented in Figure 7. The orderly matrices in the pictograph for the metal cefotaxime complexes were clarified. This demonstrated that the cefotaxime complexes had phase-material homogeneity. Many spherical black spots appeared in the cefotaxime chelates with particle sizes of 57–85, 10–15, 14–25, 16–33, and 15–25 nm for the cefotaxime complexes with Ca, Cr, Cu, Zn, and Se respectively.

### 3.8. Structures of Cefotaxime Complexes

**Figure 8:**
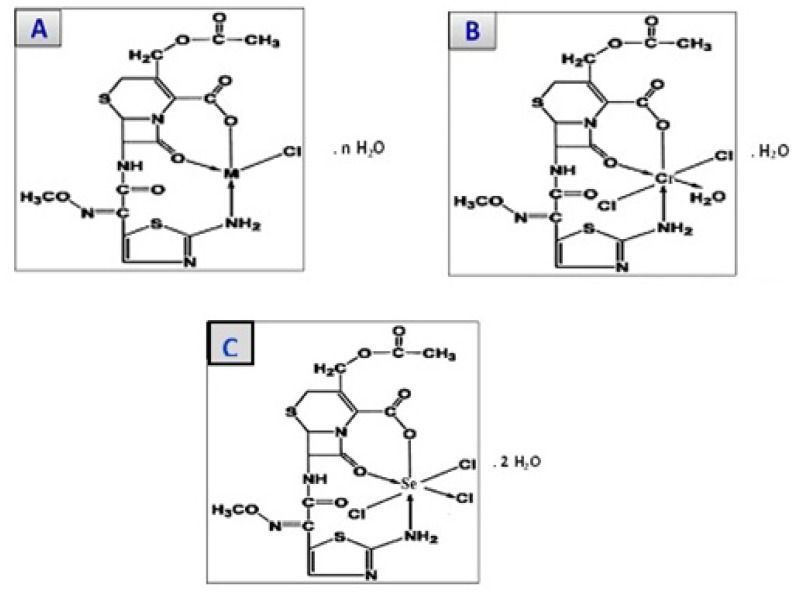
The structures of: (**A**) cefotax/M, where M = Ca (II), Cu(II), and Zn (II), and n = 1, 3, and 2, respectively; (**B**) cefotax/Cr; and (**C**) cefotax/Se.

The chemistry of the cefotaxime with the calcium, chromium, copper, zinc, and selenium metal ions was studied. Cefotaxime ions contain more than one donation atom; however, due to steric hindrance, cefotaxime can a make maximum of three donation sites (atoms) with a metal center. It has been suggested that the chelation of cefotaxime is carried out via carboxylate, amino, and lactamic carbonyl oxygen atoms. Calcium, copper, and zinc (II) complexes containing two one-chloride anions inside the complex sphere were the general formula for the [M (cefotax)Cl] complexes, where M = ca(II), cu(II), and zn(II), which were tetra-coordinated with cefotaxime as one molecule and one chloride anion. On the other hand, the chromium (III) and selenium (IV) complexes contained two and three chloride anions inside a complex sphere with an octahedral geometry. The proposed structure is shown in Figure 8.

### 3.9. Antioxidant Activities of Cefotaxime Metal Complexes

The chelating activity percentages are presented in Table 5. The ORAC assay, the metal chelation percentage, the ABTS assay, the FARAB assay, and the free-radical DPPH scavenging activities were used. The respective capacities of the cefotaxime/Cr and cefotaxime/se complexes to scavenge the free radicals were 320.02- and 303.33-fold, which were greater than that of the cefotaxime alone. Meanwhile, the metal chelating activities of the cefotaxime/Zn, cefotaxime/se, and cefotaxime/Cu were higher than that of cefotaxime alone by 15.42, 13.24, and 10.41% (µM EDTA eq/mg), respectively.

Additionally, the ferric-reducing abilities of the cefotaxime/Zn, cefotaxime/se, and cefotaxime/Cu were 179.68, 116.32, and 110.87 (µM Trolox eq/mg), respectively. These were greater than that of the cefotaxime alone, which recorded a reducing ability of 34.04 (µM Trolox eq/mg).

**Figure 9:**
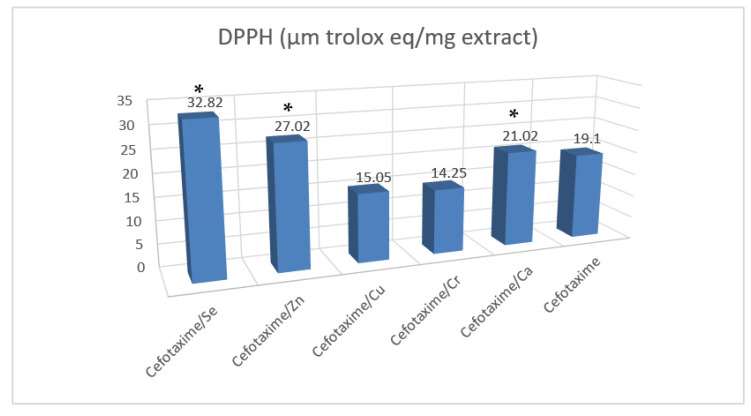

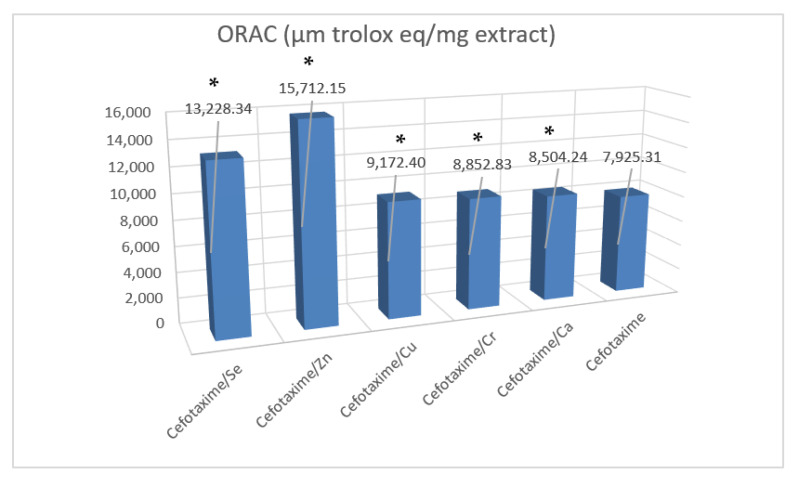
Antioxidant activity of cefotaxime and some of its metal complexes (DPPH and ORAC assays). Results are expressed as mean ± SE. (*) *p* ≤ 0.05 vs. cefotax group. Superscript * letters show significant differences (*p* < 0.05) between cefotaxime metal complexes’ activities. Trolox eq means Trolox equivalents and SD means standard deviation.

On the other hand, the scavenging abilities of both the cefotaxime/se and the cefotaxime/Zn were also the highest when measuring the DPPH stability of the radical: 32.82 and 27.02% (µM Trolox eq/mg), respectively. Thus, the cefotaxime metal complexes exhibited chelation capacities that were higher than that of cefotaxime alone (Figure 9).

The antioxidant capacities of the cefotaxime metal complexes evaluated by the oxygen radical absorbance (ORAC) are presented in Table 5 and Figure 9. However, in this case, the antioxidant activities of the cefotaxime/Zn, cefotaxime/se, and cefotaxime/Cu were 15,712.15-, 13,228.34-, and 9172.40-fold (µM Trolox eq/mg) higher, respectively, than the antioxidant activity of cefotaxime alone; these results confirmed the greater activity of the novel complexes (cefotaxime/se, cefotaxime/Zn, cefotaxime/Cu, and cefotaxime/Cr) for the absorbance of free radicals than that of the cefotaxime alone.

### 3.10. Screening of Cytotoxic Activity of Ceftox/Se and Ceftoax/Zn Metal Complexes

**Figure 10:**
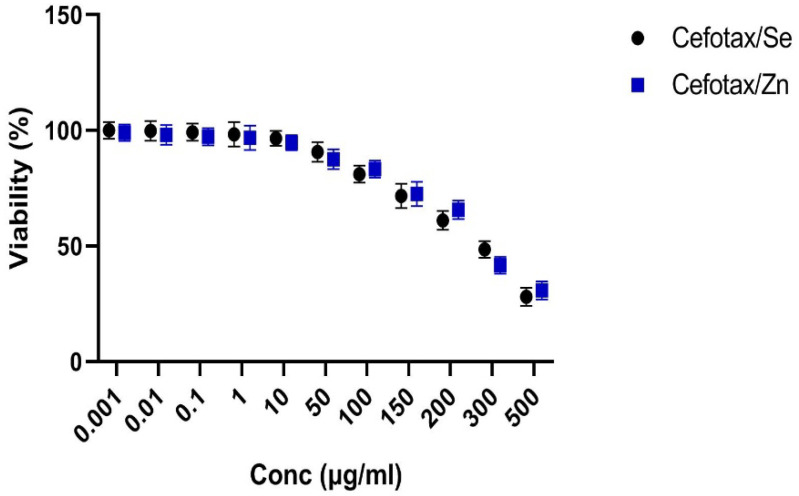
SRB (quick screening) curve of cefotax/Se and cefotax/Zn against HepG2 cells.

The Ceftox/Se and Ceftoax/Zn complexes were tested for cytotoxic activity against HepG2. The results showed that both complexes were active against HepG2; this activity differed at different concentrations, as shown in Figure 10, with an IC50 range of 200–250 µg/mL.

### 3.11. Anticancer Activity against HepG-2

**Figure 11:**
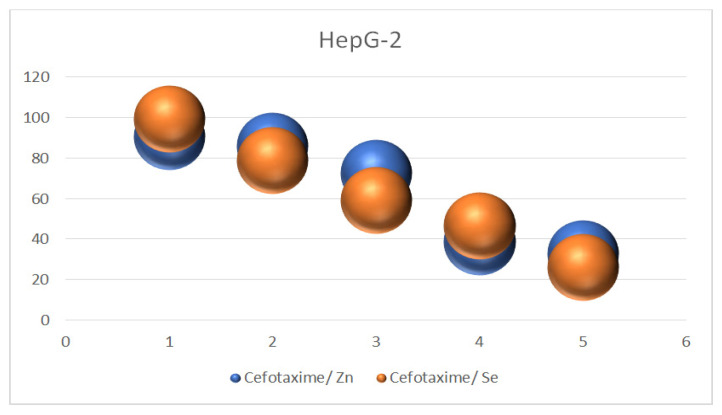
Anticancer activity of cefotaxime/Zn and cefotaxime/Se.

The application of metal drug complexes in the medical field has been confirmed for the treatment of various types of cancer cells. Cefotaxime metal complexes have been evaluated against HepG2 cancer cells. In the present study, the cytotoxic effects of cefotaxime/Se and cefotaxime/Zn were tested on HepG-2 (human hepatocellular cancer cells); the results showed that the biosynthesized cefotaxime/Se and cefotaxime/Zn demonstrated cytotoxicity against the HepG-2 cells. This was also enhanced by increasing the concentrations of the tested complexes. The inhibition of hepatocellular carcinoma growth and the decline in cellular viability in the HepG-2 cells treated with cefotaxime/Se was recorded at concentrations of 100, 200, 300, and 500 μg/mL, as follows: 81.07, 61.11, 48.52, and 28.02 µg/mL, respectively. Meanwhile, the inhibition of the hepatocellular carcinoma growth and decline in cellular viability in the HepG-2 cells treated with cefotaxime/Zn was recorded at concentrations of 100, 200, 300, and 500 µg/mL as follows: 83.25, 65.71, 41.72, and 30.82 µg/mL, respectively. These results are shown in Table 6 and Figure 11.

### 3.12. Antibacterial Activity Evaluation

**Figure 12:**
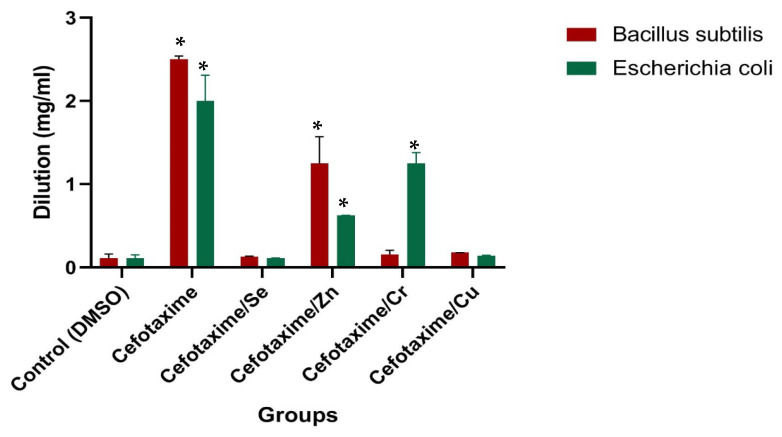
Antibacterial activity of cefotaxime and cefotaxime metal complexes, Results are expressed as mean ± SE. (*) *p* ≤ 0.05 vs. cefotax group.

**Figure 13:**
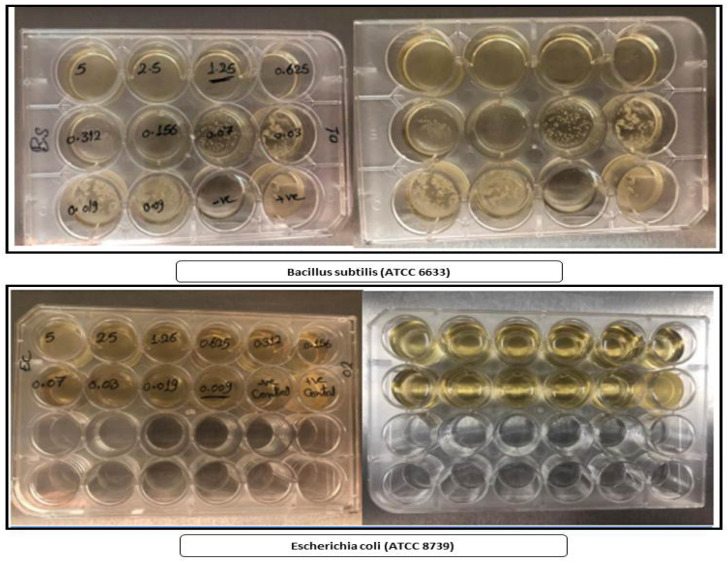
Cefotaxime and cefotaxime metal complexes vs. *Bacillus subtilis* (ATCC 6633) and *Escherichia coli* (ATCC 8739).

Biological evaluations of the target complexes were performed on Gram-positive (*Bacillus subtilis*) and Gram-negative (*Escherichia coli*) bacteria. The results of the antimicrobial activities of the cefotaxime and/or metal complexes are presented in Table 7 and Figure 12. The inhibition concentrations of the cefotaxime metal complexes (samples with the same concentration) against both Gram-positive and Gram-negative bacteria (*B. subtilis* and *E. coli*) were found to be high at very low concentrations of 0.009 and 0.03 for the cefotaxime/Se, 0.078 and 0.039 for the cefotaxime/Cu, 1.25 and 0.625 for the cefotaxime/Zn, and 0.625 and 1.25 for the cefotaxime/Cr, respectively. Based on the standard conditions, (Table 7 and Figure 12 and Figure 13), all the cefotaxime complexes were found to be sufficient, with high antimicrobial activity.

Means are expressed as (mean ± SE) and feature different letters. They are significant where *p* ≤ 0.05 using Duncan’s range test. The highest value has (a) symbol. The declining values are assigned alphabetically.

## 4. Discussion

Cefotaxime is an antibiotic drug belonging to the third generation of cephalosporins [35]; it is an active agent against both Gram-negative and Gram-positive bacterial strains via the inhibition of peptidoglycan-layer synthesis by different bacterial cellular walls [36]. The infectious diseases caused by bacterial strains are still a major health problem worldwide due to the rapid increase in bacterial resistance to existing antimicrobial drugs. Oxidative stress is also a serious problem, and it poses a high risk to human health, especially hepatic health problems [37]. Thus, the present study was conducted to prepare novel metal complexes of cefotaxime with different metal ions to clarify the antioxidant potency, antibacterial activity at lower concentrations, and hepatoprotective effects of cefotaxime complexes against HepG-2 cancer cells.

Regarding the multidrug resistance of highly pathogenic bacterial strains, the discovery of novel antimicrobial compounds is of high importance. Many microorganism strains, especially Gram-positive strains, release β-lactamase in large amounts, and they have the ability to destroy antibiotics’ β-lactam through the hydrolysis of the β-lactam rings; this is the most prevalent mechanism of antibacterial resistance [38,39].

The oxidative damage to biomolecules is of major interest in diseases such as cancer, and even serious sequences of viruses such as COVID-19. Therefore, it is urgent to examine the role of novel potent metal complexes to prevent severe oxidative injury and the treatment of diseases that involve the risk of oxidative damage.

The findings in this study confirmed the novel antioxidant capacities of cefotaxime complexes with either Se, Zn, Cr, Cu, or Ca by examining their antioxidant capacities after complexation with cefotaxime; this elevated the efficacy of cefotaxime, as it became antioxidant and antibacterial at the same time, which is very important during pandemics such as COVID-19, of which oxidative stress appears to be a catastrophic health effect, since it can destroy different bodily functions of infected patients. Thus, synthesizing a novel cefotaxime as an antibiotic drug with metals will help to alleviate oxidative injury in severe cases.

DPPH assays are used to predict the antioxidant activities through which antioxidants act to inhibit lipid oxidation, scavenge DPPH radicals and, therefore, determine the free-radical scavenging capacity [40]. The findings in this study demonstrated that cefotaxime complexes with Se, Zn, and Cu, respectively, significantly scavenged the DPPH radical, which means that the novel complexes had higher antioxidant capacities than cefotaxime alone.

Another assay that is important for the evaluation of antioxidant capacities is the ABTS assay, which measures the ability of antioxidants to scavenge the ABTS generated in the aqueous phase, compared with a Trolox (analogue vitamin E and water-soluble) standard. The ABTS is generated by reacting a strong oxidizing agent with ABTS salt [40]; our results proved that the cefotaxime complexes with Cr, Se, and Ca exhibited strong antioxidant capacities, confirming our findings.

The ORAC assay uses the ability of AAPH to form peroxyl radicals when heated in the presence of a sufficient quantity of oxygen. These radicals quench the fluorescence of a probe, thereby reducing it. The level of reduction depends on the antioxidant that quenches the produced radicals. The presented results revealed that both cefotaxime/Zn and cefotaxime/Se possessed a high capacity to reduce AAPH; thus, they possessed novel antioxidant capacities.

The liver injuries caused by many drugs may develop into chronic liver failure and hepatocellular carcinoma [41,42,43]. The majority of adverse liver responses occur due to treatments with antibiotics [44]. Several antibiotics are considered to be a common cause of the induction of hepatic damage. Hepatic impairment occurs due to hepatotoxicity [45]. The current study provided detailed results of the anticancer capacities of both cefotaxime/Zn and cefotaxime/Se in the inhibition of cancer-cell viability and a reduction to about 30 μg/mL of viable cells at a concentration of 500 (µg/mL), which is a promising sign of cefotaxime’s prospective anticancer activities.

A previous study reported that in the synthesis of cefotaxime with gold nanoparticles, cefotaxime acted as a reducing agent. The synthesized cefotaxime metal complexes were highly stable. Most importantly, they demonstrated the high antibacterial activity of cefotaxime upon loading onto the gold surfaces; at much lower concentrations, this new formula inhibited the growth of the tested bacterial strains (Gram-negative and Gram-positive) more than cefotaxime alone [46]. These findings are in agreement with those of the current study, which will expand the investigation of this novel formula with high antioxidant and antimicrobial activities.

Due to the recent increase in infectious diseases worldwide and the evolution of many resistant strains of bacteria and viruses, providing novel antibiotics with novel metals that may increase their efficacy, strength, and antioxidant activities and lower their side effects is important. The mechanism that enables bacterial strains to resist β-lactams is essentially the synthesis of β-lactamase enzymes that break the β-lactam ring; this prevents antibiotics from binding to the peptidoglycan layer [39]. We presented the novel metal complexes with cefotaxime and examined their biological activity against both *Bacillus s.* and *Escherichia c.* stains; they had high antibacterial activity against them at very low concentrations, which is a promising result. Previous studies reported the antimicrobial activity of different cephalosporins strains after complexation with metal complexes, confirming our findings [47,48,49].

## 5. Conclusions

The present study prepared five cefotaxime metal complexes and chemically characterized the products of the reaction of cefotaxime with Ca^2+^, Cr^+3^, Cu^2+^, Zn^2+^, and Se^4+^ ions. The cefotaxime complexes’ structures were characterized through elemental analysis, molar conductance, FTIR, ^1^HNMR, electronic spectra, magnetic, scanning and transmission electron microscopy, and X-ray diffraction analyses. The Cr(III) and Se(VI) complexes formed a coordination number equal to six, with distorted octahedral geometry. The acquired results showed that the cefotaxime metal complexes (Cefotax/Zn and Cefotax/Se) greatly inhibited the hepatocellular carcinoma viability percentage in HepG-2 cells and, with other complexes of cefotax/Cr, cefotax/Cu, and cefotax/Ca, enhanced the antioxidant activities tested with DPPH, ABTS, ORAC, FARAB, and metal chelation assays compared with cefotaxime alone. For antibacterial activity, the cefotaxime metal complexes were effective against bacterial strains of *Bacillus subtilis* and *Escherichia coli*. The obtained results are very promising, since they demonstrate the provision of strong protection against hepatocellular carcinoma (HepG-2) and a reduction in the damaging effects and the severe oxidative stress induced by antibiotics, especially during the COVID-19 pandemic.

## Figures and Tables

**Table 1:** Physical characterization and micro-analytical and molar conductance data of cefotaxime complexes.

Complexes	M. Wt			Elemental Analysis (%)		Λm (Ω^−1^cm^2^mol^−1^)	
		C	H	N	S	M^+n^	
[Ca(Cefotax) Cl]·H_2_O	565.50	(33.95) 33.88	(3.53) 3.46	(12.37) 12.64	(11.31) 11.74	(7.08) 6.89	18
[Cr(Cefotax) (H_2_O) (Cl)_2_]·H_2_O	612	(31.37) 31.84	(3.26) 3.85	(11.43) 11.23	(10.45) 10.92	(8.49) 8.35	23
[Cu(Cefotax) Cl]·3H_2_O	607	(31.63) 31.63	(3.62) 3.64	(11.53) 11.83	(10.54) 10.71	(10.46) 11.03	16
[Zn(Cefotax) Cl]·2H_2_O	589	(32.59) 32.72	(3.39) 3.89	(11.88) 11.29	(10.86) 10.45	(11.10) 11.51	17
[Se(Cefotax) (Cl)_3_]·2H_2_O	675.50	(28.42) 28.59	(2.96) 3.31	(10.36) 10.83	(9.47) 9.93	(11.69) 11.32	24

**Table 2:** Infrared assignments of cefotaxime and its metal complexes.

Assignments		Complexes				
	Cefotax	Ca(II)	Cr(III)	Cu(II)	Zn(II)	Se(IV)
ν(O-H), COOH	3360	--	--	--	--	--
ν(O-H), H_2_O	--	3335	3360	3334	3345	3365
ν(N-H), NH_2_	3330	3310	3252	3316	3208	3230
ν(C-H); Aromatic	3075	3064	3070	3082	3071	3067
ν(C-H); Aliphatic	2938	2945	2941	2925	2936	2929
ν(C=O), lactam	1775	1720	1728	1715	1733	1740
ν(C=O)ester + ν(C=O)amide	1642	1650	1645	1630	1640	1646
ν_as_ (COO^−^)	1598	1606	1607	1600	1612	1604
ν_s_ (COO^−^)	1380	1385	1385	1384	1390	1375
∆ν = ν_as_ (COO^−^)– ν_s_ (COO^−^)	218	221	222	216	222	229
ν (M-O)	--	640 530	618 518	624 523	618 525	614 536
ν (M-N)	--	470	476	495	480	475

**Table 3:** UV–vis spectra of cefotaxime and its metal complexes.

Compound	π → π*	n → π*
Cefotax	273,320	350
Ca(II)	285,322	391
Cr(III)	280,327	405
Zn(II)	288,331	407
Se(IV)	286,340	410

**Table 4:** ^1^H-NMR assignments for free-ligand cefotaxime and its metal complexes.

^1^H-NMR Assignments		δ(ppm)		
	Cefotaxime	Ca(II)	Zn(II)	Se(VI)
1H; COOH	12	-	-	-
6H; 2CH_3_	1.851	1.864	1.894	1.892
2H; S-CH_2_	3.127	3.129	3.131	3.165
2H; O-CH_2_	4.741	4.807	4.762	4.745
1H; CO-CH	4.956	4.989	4.975	4.964
1H; N-CH	5.770	5.617	5.575	5.589
2H; NH_2_	7.459	7.249	7.255	7.229
1H; NH	9.534	9.535	9.538	9.532
2H; H_2_O	-	3.831	3.836	3.808

**Table 5:** Antioxidant activity of cefotaxime and its complexes (cefotaxime/Ca, cefotaxime/Cr, cefotaxime/Cu, cefotaxime/Zn, cefotaxime/Mg, and cefotaxime/Se).

Test Name	ORAC		Metal Chelation		ABTS		FARAB		DPPH	
Sample Name	(µM Trolox eq/mg)		(µM EDTA eq/mg)		(µM Trolox eq/mg)		(µM Trolox eq/mg)		(µM Trolox eq/mg)	
	Mean	SD	Mean	SD	Mean	SD	Mean	SD	Mean	SD
cefotaxime	7925.31 ^e^	363.96	2.99 ^e^	0.59	288.52 ^d^	12.26	34.04 ^e^	3.72	19.10 ^d^	0.29
cefotaxime/Ca	8504.24 ^d^	378.36	2.98 ^e^	0.34	296.02 ^c^	4.58	24.05 ^f^	1.01	21.02 ^c^	3.25
cefotaxime/Cr	8852.83 ^d^	471.45	6.42 ^d^	0.84	320.02 ^ab^	3.25	106.02 ^d^	9.58	14.25 ^e^	2.40
cefotaxime/Cu	9172.406 ^c^	532.59	10.41 ^c^	2.96	289.14 ^d^	4.69	110.87 ^c^	10.69	15.05 ^e^	1.69
cefotaxime/Zn	15,712.15 ^a^	354.84	15.42 ^a^	2.52	278.02 ^e^	5.02	179.68 ^a^	4.58	27.02 ^b^	1.58
cefotaxime/Se	13,228.34 ^b^	734.90	13.24 ^b^	0.24	303.33 ^b^	2.64	116.32 ^b^	11.16	32.82 ^a^	2.05

Results are expressed as mean ± SD. Symbols are different alphabetically (a–f) to indicate a significant comparison compared to the control group and other treated groups (*p* < 0.05) (Similar letters imply partial or complete non-significance).

**Table 6:** Anticancer activity of cefotaxime and its metal complexes (cefotaxime/Se, and cefotaxime/Zn).

HepG-2				
Viability %	Cefotaxime/Se (µg/mL)		Cefotaxime/Zn (µg/mL)	
	Mean	SD	Mean	SD
10 μg/mL	96.52	2.05	94.70	1.42
100 μg/mL	81.07	1.06	83.25	0.11
200 μg/mL	61.11	2.60	65.71	2.41
300 μg/mL	48.52	1.80	41.72	2.01
500 μg/mL	28.02	2.68	30.82	1.97

**Table 7:** Dilution concentration (mg/mL sample) and bacterial inhibition concentration of cefotaxime and its metal complexes (three replicates).

Sample	Dilution (mg/mL)	
	*Bacillus subtilis* (G^+^)	*Escherichia coli* (G^−^)
Control (DMSO)	0.0 ± 0.00 ^e^	0.0 ± 0.00 ^f^
Cefotaxime	2.5 ± 0.04 ^a^	2.0 ± 0.31 ^a^
Cefotaxime/Se	0.03 ± 0.007 ^d^	0.009 ± 0.004 ^e^
Cefotaxime/Zn	1.25 ± 0.32 ^b^	0.625 ± 0.001 ^c^
Cefotaxime/Cr	0.156 ± 0.05 ^c^	1.25 ± 0.13 ^b^
Cefotaxime/Cu	0.078 ± 0.001 ^c^	0.039 ± 0.007 ^d^

Means are expressed as (mean ± SE) and feature different letters. They are significant where *p* ≤ 0.05 using Duncan’s range test. The highest value has (a) symbol. The declining values are assigned alphabetically.

## Data Availability

All the Data are available within the text.
